# PTSD in the military: special considerations for understanding prevalence, pathophysiology and treatment following deployment

**DOI:** 10.3402/ejpt.v5.25322

**Published:** 2014-08-14

**Authors:** Rachel Yehuda, Eric Vermetten, Alexander C. McFarlane, Amy Lehrner

**Affiliations:** 1 James. J. Peters Veterans Affairs Medical Center, New York, NY, USA; 2 Icahn School of Medicine at Mount Sinai, New York, NY, USA; 3 Department of Psychiatry, Leiden University Medical Center, Leiden, The Netherlands; 4 Arq Psychotrauma Expert Group, Diemen, The Netherlands; 5 Military Mental Health Research, Department of Defense, Utrecht, The Netherlands; 6 Centre for Traumatic Stress Studies, The University of Adelaide, Adelaide, South Australia, Australia

**Keywords:** posttraumatic stress disorder, combat, military, deployment, prevalence, treatment, biomarkers

## Abstract

Given the unique context of warzone engagement, which may include chronic threat, multiple and lengthy deployments, and loss, there is a need to understand whether and to what extent knowledge about PTSD derived from studies of civilian trauma exposure is generalizeable to the military. This special issue on PTSD in the military addresses a range of issues and debates related to mental health in military personnel and combat veterans. This article provides an overview of the issues covered in selected contributions that have been assembled for a special volume to consider issues unique to the military. Several leading scholars and military experts have contributed papers regarding: 1) prevalence rates of PTSD and other post-deployment mental health problems in different NATO countries, 2) the search for biomarkers of PTSD and the potential applications of such findings, and 3) prevention and intervention approaches for service members and veterans. The volume includes studies that highlight the divergence in prevalence rates of PTSD and other post-deployment mental health problems across nations and that discuss potential causes and implications. Included studies also provide an overview of research conducted in military or Veteran's Affairs settings, and overarching reviews of military-wide approaches to research, promotion of resilience, and mental health interventions in the Unites States and across NATO and allied ISAF partners.

In this issue, we have brought together researchers and strategic thinkers involved in topics relevant to military mental health. We address three important areas: 1) the prevalence rates of posttraumatic stress disorder (PTSD) and other mental health problems between nations, 2) the potential identification of biological markers of PTSD or other deployment-related mental health problems, and 3) interventions for military personnel and combat veterans. The decision to compile a special issue focusing on PTSD issues relevant to the military was catalyzed by a need to examine the generalizability and relevance of findings from civilian populations to the military context. The unique nature of combat and deployment-related trauma, including lengthy and repeated deployments, chronic threat, and multiple trauma exposures, may have different biological and psychological implications compared to civilian traumas.

Deployment-related PTSD, as well as associated mental health problems (e.g., depression, alcohol and drug abuse) presents a major challenge to military and veteran treatment facilities worldwide. The burden associated with these problems, including human suffering, lost productivity, and disability, is substantial (Deahl, Klein, & Alexander, 2011; Sabes-Figuera et al., 2012). While advancements have been made over the past few decades in understanding and treating symptoms of PTSD, at the writing of this issue there remain significant gaps in knowledge and several unresolved issues. As highlighted by this issue, there has been fierce disagreement regarding the prevalence of PTSD following deployment among different militaries around the world, making it difficult to fully comprehend the relationship between combat exposure and the development of mental health symptoms after deployment. Gains in understanding biological aspects of PTSD have also been consistently made, and there is now growing convergence regarding the major brain, neurochemical and neuroendocrine systems involved in deployment-related injuries such as PTSD.

A critical question, however, is whether and to what extent biological and other aspects of PTSD resulting from deployment are equivalent to PTSD that occurs in civilian contexts. Clinical and biological studies of PTSD tend to focus on commonalities in presentations across different populations of trauma survivors, while far less often emphasizing specific consequences of particular trauma types. A majority of studies include a heterogeneous group of persons that meet the diagnostic criteria for PTSD at the time of study, generally without consideration of trauma type (e.g., exposure to interpersonal violence, natural disaster, accident, or combat). While the focus on similarities among trauma survivors yields important information, investigations of variation as a result of trauma type might also bear fruit. There is also an increasing interest in understanding how characteristics of different combat theatres may contribute to new and unique clinical presentations.

Although warfare has occurred since early recorded history, its characteristics have changed dramatically with the ever increasing complexity of technology (Singh & Sharma, 2013; Sutherland, 2012). First and foremost, war between nations is now largely asymmetric. Thus, opposing nations frequently have unequal military resources with undefined battlefields, where terror tactics affect civilians and military personnel alike. Advances in technology have further changed the type of casualties sustained. The nature of modern warfare no longer results in the mass battlefield casualties of earlier conflicts such as the First and Second World Wars and the Vietnam War. As more soldiers survive deployment-related experiences, attention must be paid to the profound transformations resulting from deployment, including invisible, psychic wounds.

Those who work with military personnel or veterans are highly attuned to the ways in which such persons differ from traumatized civilians. Many who choose military service, and even many of those who are mandated to serve, embrace both the larger principals of duty to country and the day-to-day regimens that result in a deep connection and loyalty to fellow service members. Warfighters also receive extensive training and preparation for combat trauma. Upon return from deployment, even in cases where the warfighter had been exposed to one or more life-threatening or horrifying experience and prolonged periods of threat in malevolent environments, many soldiers regard the deployment experience as the best and the worst of times, and some even seek to return to the battlefield. Obviously, these latter characteristics distinguish combat veterans from traumatized adult civilians who do not generally consciously seek to return to the scene of a trauma, with the exception of members of the emergency services whose exposures have much in common with military personnel. The social connections formed to fellow servicemen are often difficult to break, and the necessity of severing such connections to return home to reintegrate to a pre-deployment environment are also quite challenging, and form an unusual backdrop for PTSD symptoms.

The question of whether biological and other aspects of PTSD resulting from deployment are equivalent to civilian PTSD is particularly important in designing specialized treatments for service personnel and veterans. At the current time, treatments for combat-related PTSD guarantee neither significant nor sustained improvement. A substantial proportion of veterans with PTSD and related mental health conditions receive treatment for years or even decades. At the same time, despite some improvements in recent years, many warfighters still are reluctant to seek care, and face barriers to care and stigma. The nature of combat, which may involve killing, witnessing scenes of death and dismemberment, and difficult split-second decisions, may also distinguish post-deployment mental health sequelae and require specialized pre-deployment training, as suggested by Thompson and Jetly (2014) in this issue, as well as specialized treatments that address these complex issues.

We have invited colleagues from a wide range of settings to compile information that is rarely considered in academic volumes because it is derived from internal inquiries to help support decision-making within and between nations. While some of the information obtained does not follow the same methodology or conventions, persons who collect data in the context of informing military operations often have access to information that is otherwise not easily obtained. It is important to bring such information to the attention of academics and clinicians, in addition to more traditional research papers or clinical case studies. We focus in this issue on research derived or sponsored by militaries or Veterans Affairs, and on the extent to which the resultant knowledge is synergistic with information obtained in parallel from civilian settings.

## Different rates of PTSD and other mental health problems between nations: real or illusory?

The first set of papers report findings of epidemiological studies in different militaries (Hunt, Wessely, Jones, Rona, & Greenberg, 2014; Taal, Vermetten, Digna, van Schaik, & Leenstra, 2014; Van Hooff et al., 2014; Zamorski & Boulos, 2014). These papers show that estimates of the prevalence of mental health consequences (e.g., PTSD, suicidality, mild TBI, and other deployment-related conditions) vary drastically both across and within nations. For example, it can be clearly seen in Hunt et al. (2014) that the prevalence of reported PTSD is lower in the United Kingdom armed forces than is reported in countries such as the United States, Australia, and Canada (Castro, 2014; Van Hooff et al., 2014; Zamorski & Boulos 2014, all this issue). It is also clear that different sampling strategies (including cohort and timeframe), methods and thresholds for diagnosis, and consideration of additional risk factors, such as age and combat experiences, yield markedly different prevalence estimates and make cross-country comparisons difficult.

Hunt et al. (2014) review the divergent prevalence findings between the UK and other allied nations and discuss possible explanations. PTSD rates in British forces are estimated at roughly 4% in personnel who have deployed, and 6% in combat troops, and they note high levels of alcohol abuse and increased rates of violence among post-deployment UK combat forces. The authors provide an extensive discussion of factors in addition to those noted above that may affect UK prevalence estimates, including differences in combat exposure, demographics, tour length, troop structure (vs. the US), the nature and non-anonymity of assessment, and access to post service universal health care. Zamorski & Boulos (2014) review all major epidemiological studies of mental health outcomes before and during the Afghanistan era in Canadian Armed Forces. The authors note that such studies are few, with inadequate detail about deployment experiences. Their review also highlights how different methods and samples yield strikingly different findings, even within one country. Canadian estimates of post-deployment PTSD range from 8 to 20% across studies. They find that combat exposure is the most important factor for deployment-related mental health problems.

Van Hooff et al. (2014) describe the design, sampling strategies, and methodology of an ambitious epidemiological study of the prevalence of mental disorders in the Australian Defence Force (ADF), the ADF Mental Health Prevalence and Wellbeing Study (MHPWS). This investigation represents the first prevalence study in an entire military population, and includes psychiatric interviews, a significant improvement over chart review or self-report data. Findings indicate that 22% of ADF members had a mental health disorder in the prior 12 months, the most common of which were anxiety disorders (15%). Taal et al. (2014) analyzed mental health consumption for the full cohort of enlisted armed forces in the Netherlands between 2008 and 2010. The authors note that warfighters reported being reluctant to seek care, perceived barriers to care and feared the impact of stigma. Nonetheless, they do find their way to treatment. Interestingly, there was a five-fold increase in PTSD diagnoses in the first 2 years following deployment to Afghanistan, which may be interpreted as reflecting delays in accessing care, the development of delayed onset PTSD, or a gradual increase in the interference of symptoms in everyday life. An important issue raised by this paper is the extent to which the development of adequate mental health infrastructures should depend on the proportion or absolute number of warfighters affected. The authors suggest that the figures from the Netherlands do not support the view presented in the media that military involvement in Afghanistan has led to an “epidemic of psychiatric illnesses,” but that the escalating symptoms over a 5-year period justify the development of a strong mental health infrastructure for returning soldiers.

In addition to the methodological issues noted above, it is important to consider potential structural factors that may influence estimates of post-deployment or service mental health problems. For example, unlike the other countries mentioned above, the United Kingdom does not have a veterans, system of health care. It is possible that the problem of PTSD in the military might be less detectible because once individuals leave Defense in the United Kingdom, their ongoing health care becomes the responsibility of the National Health System, and is therefore harder to track as a military-related problem. It is certainly the case that to the extent that there are long-term consequences associated with war, this would represent a major forward liability for any government to fund, particularly in terms of pensions and health costs. In cases such as the United Kingdom, where this cost is hidden within the much larger national health budget, its relevance and significance is not subject to actuarial scrutiny, and the burden of military service may be underestimated.

Thus, epidemiological research on PTSD and other post-deployment mental health conditions has significant budgetary and political, as well as scientific, consequences. When reported prevalence rates are low, it is more difficult to conceptualize post-deployment mental health symptoms as war wounds, and such findings may be used as justification for the lack of an integrated veterans’ health system. Indeed, countries which do not consider mental health outcomes as a cost of war may not make appropriate provisions for treatment. Furthermore, such governments are also not accepting liability associated with having contributed to long-term disability in troops sent to war. When there is a higher prevalence it is easier to conclude that warzone experiences represent the major causal antecedents or precipitants of illness, and such findings provide support for investments in research, prevention, treatment, and longer-term care for soldiers and veterans.

Despite the major policy, fiscal, and programmatic implications of prevalence estimates of deployment-related mental health problems, the significant methodological differences across studies are seldom highlighted in the debate surrounding international comparisons of post-deployment morbidity. Clearly rates are higher in studies that use anonymous self-report questionnaires rather than structured interviews, and in investigations that compare deployed with non-deployed groups (Hoge et al., 2004). Furthermore, longitudinal studies of the US military on the impact of deployment, which permit individuals to act as their own controls, generally do point to significant rates of morbidity as a consequence of deployment (Tanielian & Jaycox, 2008). A recent publication demonstrated that the dramatic differences in estimates of the psychological burden of combat between the United States and the United Kingdom largely disappear once self-reported combat exposure is taken into account (Sundin et al., 2014).

## Biomarkers of PTSD

Given the methodological and structural challenges in establishing valid prevalence rates that can be compared across nations, the potential identification of biological markers of PTSD or other deployment-related mental health problems offers alternative, objective prevalence measures. There has been an enormous effort to capture risk and resilience factors, as well as identify biomarkers of expressed illness (Yehuda, Neylan, Flory, & McFarlane, 2013). Three papers in this issue highlight some of the important developments in identifying biomarkers of risk and resilience (Daskalakis & Yehuda, 2014; Lehrner & Yehuda, 2014; Neylan, Schacht, Yehuda, 2014). Neylan et al. (2014), review the need to identify illness biomarkers and the various approaches that should be used. The development of numerous biological methods that permit large-scale screening of biomolecules (genome-wide genotype, epigenetic, gene expression, and large numbers of proteins and metabolites), as well as computational advancements, leave the field well poised to identify biomarkers using discovery-based approaches. In the past, progress has been impeded by the necessity of developing elaborate models and narrowly focusing on specific markers and systems. Hypothesis generating approaches were previously viewed as expensive “fishing expeditions” that increased the probability of false positives. However, sophisticated procedures for computation and validation of discovered biomarkers have reduced these concerns and heralded a new era, which promises the delivery of objective biological indicators of a mental battlefield injury.

While it is universally recognized that modern medicine would be seriously constrained without diagnostic blood tests, biopsies, and imaging, psychiatry and mental health have remained largely without objective diagnostic tests or prognostic indicators. It is important to consider how the availability of such markers would affect soldiers. It is reasonable to assume that for many, not having validation of PTSD as a legitimate war-related injury fuels the perception that PTSD and other deployment-related mental injuries are not “real.” The fact that only some combat soldiers develop PTSD may be seen by some as a reflection of personal weakness rather than of the life-altering impact of trauma. This stigma deepens the abyss between warfighters and the social support networks crucial to their recovery. The delivery of objective biological indicators of a mental battlefield injury, or a test that would facilitate engagement in treatment early on in the progression of the disease, might preempt worsening of illness and the deleterious interpersonal consequences that occur when initial symptoms are neglected and avoided. Biomarkers that could reflect fitness for duty following a combat injury would also be important, by helping the military to make reasonable policy decisions regarding who is fit to serve even in the context of past mental health symptoms. This is important because an overly conservative stance that prevents further combat exposure ostensibly to protect combat veterans with prior illness may be stigmatizing and result in a reluctance to report symptoms. Alternatively, for those that believe they are more impaired than they in fact may be, an objective measure may provide information that can allow soldiers to mobilize resilience-related resources and return to military duty and/or occupational functioning post-deployment.

In the push to identify PTSD biomarkers, there has been little discussion of how these markers will be used in clinical and non-clinical settings, as well as the legal and ethical implications. Even before troops are deployed, how will the military use biomarkers to screen, hire, place, or even reject potential recruits? Once they are deployed or return from deployment, can biomarkers be used to diagnose acute or chronic combat-related illnesses or determine fitness for duty or disability? Lehrner and Yehuda (2014) discuss how biomarkers might be used in the context of prediction of risk prior to deployment, diagnosis, prognosis, and triage in the military. They also discuss potential unintended consequences of the use of biomarkers and the need for more engaged dialogue in the field and with legal and ethics experts.

Daskalakis and Yehuda (2014) discuss the relevance of animal studies to the identification of combat-related biomarkers. In animal literature there is a great deal of attention on the “nature” of the stress exposures. In contrast, in human research relatively little attention is paid to the highly diverse nature of traumatic experiences, arguably a critical factor in human variation in response to trauma exposure. Insofar as animal studies hold potential to advance our understanding of PTSD, it is important to consider differences in biological findings when comparing trauma or stress exposed to non-exposed animals, versus those that result from examining individual differences. This paper also makes a contribution in examining how findings from animal models of PTSD are critical to advancing efforts in clinical treatment. Ideally, information from blood and brain from humans and animals, carefully considered in tandem and possibly even computed simultaneously, can be used to identify molecules, pathways and networks that are likely to be the key drivers of PTSD symptoms. With animal models, and newer biological methodologies, critical genes and pathways can be tuned up or down (rather than ablated completely) in discrete brain regions. Such techniques in tandem with human imaging and blood studies will accelerate the identification of novel pharmacological and non-pharmacological intervention strategies.

## Interventions for military personnel and combat veterans

A notable finding in the PTSD treatment outcome literature has been that randomized clinical trials often have better outcomes in civilians than in combat veterans (Bradley, Greene, Russ, Dutra, & Westen, 2005). This has been demonstrated for both pharmacological and psychotherapeutic studies. In fact, in industry-sponsored clinical pharmacological trials conducted in the late 1990s, combat veterans receiving treatment at US Veterans Administration (VA) medical centers were excluded because of the concern that including such patients might result in falsely negative findings. It remains a question whether the kind of treatment regimen that has worked well for civilians is adequate for treating the specific issues that result from combat exposure. There are also concerns about the high dropout rates for evidence-based CBT treatments at the VA, and the small effect sizes noted for treatment completers with these treatments. As most specialized therapies for PTSD require engaging with the trauma memory and associated emotions, it may be particularly difficult for military veterans to engage in these treatments given their training on self-discipline, self-control, and hypervigilance. Furthermore, for veterans who have experienced loss, guilt, or shame, it is not clear whether exposure-based therapies are effective. There are additional outcomes above and beyond those associated with PTSD that are particularly relevant for warfighters. In addition to focusing on core PTSD symptoms, it is appropriate to consider strategies for helping combat veterans with memory and cognition, ability to function in school or work, tolerance of affect and emotions in general, effective parenting, and overall mental and physical health.

Thompson and Jetly (2014) describe a novel program for moral dilemma training prior to deployment, which could help reduce battlefield stressors and ethical lapses. They propose adjunctive scenario-based ethics training in the field in support of conventional military ethics training and education. Such preparation may serve to enhance psychological resilience and wellbeing during and after deployment as well as improve the effectiveness of the military mission. It is particularly important that the military is aware of the need to provide such training, as there are often highly ambiguous situations during combat. The recognition that the experience of having participated in war—however justified and morally executed—may create an existential crises is a significant advance.

Ivanov and Yehuda (2014) discuss the challenges to differential diagnosis posed by comorbid attention deficit hyperactivity disorder (ADHD) and PTSD symptoms, and the relevance of ADHD for military service and deployment. They suggest that ADHD may differentially affect fitness for service versus deployment, which raises similar questions about screening, job placement, deployment, and exposure to potential stressors to those noted above regarding the use of mental health biomarkers in military contexts. They also show how the unique neurobiology of these disorders complicates complementary pharmacologic treatment, as standard treatment for one disorder (e.g., stimulants for ADHD) may exacerbate the other (e.g., cause increased anxiety and agitation in PTSD).

Vermetten et al. (2014) provide a novel, broad-ranging, comparative analysis of approaches to mental health care across NATO and allied ISAF partners including the United Kingdom, Canada, Australia, the United States, and the Netherlands. This group of investigators, all participants in NATO sponsored panels, discuss issues that may account for the different prevalence estimates across and within nations. The review summarizes ingredients of state-of-the-art preventative mental health care. To further reduce the prevailing stigma around mental problems, training for ‘social leadership’ and ‘paraprofessional peer supporters’ were identified as key important topics across surveyed countries. Both approaches may speed responses to mental health issues within the unit, lowering dependency on scarce mental health professionals.

Castro (2014) provides a bird's eye view of the US Department of Defense (DoD) approach to mental health for veterans of the conflicts in Iraq and Afghanistan. This paper describes the DoD's Psychological Health Research Continuum, which guides the research strategy for PTSD, mTBI, and suicide. The Continuum specifies research that will support “understanding, prevention, and intervention,” which includes basic science, epidemiology, etiology, prevention and screening, treatment, follow up care, and systems of care. This paper also reviews issues regarding combat and mental health, including risk and resilience factors of mental health, biomarkers of PTSD, mental health training, psychological screening, psychological debriefing, third location decompression, combat and suicide, psychotherapy and drug therapy for PTSD, the role of advanced technology, telemedicine and virtual reality, methods to reduce stigma and barriers to care, and best approaches to disseminate effective interventions. Finally, a brief review and discussion of the research looking at special populations, including National Guardsmen and reservists, female service members, and ethnicity and race, and the military family is provided.

## Conclusion

Much of the knowledge that has accrued regarding the mental health consequences of war has been generated by the military, but increased funding has also supported research in a variety of clinical and academic settings. Studies documenting the prevalence of PTSD and other mental health consequences of war have had the most variable results, and the studies in this issue highlight some of the implications associated with different reported rates of PTSD following combat.

In parallel with epidemiologic studies documenting the prevalence of mental health problems, there is an effort to capture risk and resilience factors, as well as to identify biomarkers of expressed illness. Such biologically informed studies may ultimately provide relevant data regarding prevalence. That is, to the extent that soldiers can submit to a single blood test that informs their mental health symptoms, this potentially provides an important context for interpreting the nature of combat wounds. Previously, there have been limited resources for examination of biomarkers as either risk factors or diagnostic markers in the military, but the growing trend is to investigate biological factors that may inform clinical and occupational decision-making.

Finally, both epidemiologic and biologic studies are performed to elaborate the scope and specifics of post-deployment mental health problems and to promote prevention and treatment interventions. There are several important issues here. The first concerns whether combat-related mental health consequences are similar to those associated with other traumatic exposures. For example, there has been an implicit assumption in the field that with respect to PTSD, combat is a precipitating traumatic event similar to other forms of interpersonal violence. However, in conceptualizing resilience training and other prophylactic strategies, as well as immediate and long-term mental health treatment for war veterans, it is important to be certain whether combat trauma is similar to other traumatic events that are associated with an extreme fear response. Understanding some of the specific demands and moral conflicts of the battlefield will help direct the field toward more nuanced investigations into disease risk and etiology and the development of more specific treatments.
